# Monocytes Expose Factor XIII-A and Stabilize Thrombi against Fibrinolytic Degradation

**DOI:** 10.3390/ijms22126591

**Published:** 2021-06-19

**Authors:** Fahad S. M. Alshehri, Claire S. Whyte, Ahmet Tuncay, Maria-Louise Williams, Heather M. Wilson, Nicola J. Mutch

**Affiliations:** Aberdeen Cardiovascular & Diabetes Centre, Institute of Medical Sciences, University of Aberdeen, Aberdeen AB25 2ZD, UK; r01fsma@abdn.ac.uk (F.S.M.A.); c.s.whyte@abdn.ac.uk (C.S.W.); 17029677@uhi.ac.uk (A.T.); r01mw16@abdn.ac.uk (M.-L.W.); h.m.wilson@abdn.ac.uk (H.M.W.)

**Keywords:** factor XIII-A, fibrinolysis, monocytes, thrombi, transglutaminase, cross-linking

## Abstract

Factor XIII (FXIII) is a transglutaminase that promotes thrombus stability by cross-linking fibrin. The cellular form, a homodimer of the A subunits, denoted FXIII-A, lacks a classical signal peptide for its release; however, we have shown that it is exposed on activated platelets. Here we addressed whether monocytes expose intracellular FXIII-A in response to stimuli. Using flow cytometry, we demonstrate that FXIII-A antigen and activity are up-regulated on human monocytes in response to stimulation by IL-4 and IL-10. Higher basal levels of the FXIII-A antigen were noted on the membrane of the monocytic cell line THP-1, but activity was significantly enhanced following stimulation with IL-4 and IL-10. In contrast, treatment with lipopolysaccharide did not upregulate exposure of FXIII-A in THP-1 cells. Quantification of the FXIII-A activity revealed a significant increase in THP-1 cells in total cell lysates following stimulation with IL-4 and IL-10. Following fractionation, the largest pool of FXIII-A was membrane associated. Monocytes were actively incorporated into the fibrin mesh of model thrombi. We found that stimulation of monocytes and THP-1 cells with IL-4 and IL-10 stabilized FXIII-depleted thrombi against fibrinolytic degradation, via a transglutaminase-dependent mechanism. Our data suggest that monocyte-derived FXIII-A externalized in response to stimuli participates in thrombus stabilization.

## 1. Introduction

The protransglutaminase factor XIII circulates in plasma (pFXIII) as a tetramer of two A subunits and two carrier B subunits [1]. It is activated at the final stages of the clotting reaction via the concerted action of thrombin and Ca^2+^. The resulting active enzyme, pFXIIIa, catalyzes an acyl transfer reaction between lysine and glutamine amino acids forming intra- or inter-molecular cross-links [1]. The primary substrates for pFXIIIa are fibrin and α_2_antiplasmin (α2AP), cross-linking of which increases the stability of a fibrin clot against mechanical stress and fibrinolytic degradation, respectively [2]. A deficiency in FXIII manifests as a severe bleeding diathesis with patients requiring regular FXIII replacement therapy. In addition to its essential role in hemostasis, pFXIII functions in several other related biological processes, such as the maintenance of pregnancy [1], wound healing and angiogenesis [3].

The cellular form of FXIII is a homodimer of the A-subunits, termed FXIII-A that is localized to a wide variety of cells, including platelets, megakaryocytes, monocytes, and tissue macrophages, dendritic cells, chondrocytes, osteoblasts and preadipocytes (recently reviewed by [4]). Cellular FXIII-A is non-proteolytically activated by modest increases in intracellular Ca^2+^ concentrations [5,6]. The mechanism of FXIII-A release from these cells remains an enigma, as it lacks a signal sequence and is absent from the endoplasmic reticulum (ER)-Golgi secretory pathway in nucleated cells [7]. However, FXIII-A in monocyte-macrophages is reportedly directed to the plasma membrane in association with Golgi vesicles [8] indicating secretion follows an alternative pathway. 

Platelets were hypothesized to be the source of the FXIII-A subunit in plasma [9,10] as they contain an abundance of FXIII-A in their cytoplasm. However, this was ruled out, as in thrombocytopenic mice plasma levels of FXIII-A were within the normal range [8]. Tissue-specific mouse knockouts of FXIII-A pinpoint resident tissue macrophages as the cellular source of plasma FXIII-A [11]. Nonetheless, our laboratory has demonstrated that activated platelets expose functional FXIII-A which is capable of crosslinking α_2_AP and fibrin, therefore stabilizing thrombi against degradation [12]. The platelet pool of FXIII-A may be functional in the microenvironment of the thrombus where solute transport of the plasma pool is low [13]. 

There is mounting evidence that venous thrombosis is an inflammatory condition and in line with this, acute infections predispose to the condition [14]. Monocytes have been proposed to function in the initiation and propagation of venous thrombosis [15] and are actively recruited to thrombi in a cytokine-dependent manner [16]. Monocytes are a rich source of FXIII-A [17]; however, as it is not classically secreted it is unclear whether this transglutaminase could participate in stabilization of venous thrombi. In this study we investigate whether FXIII-A is externalized on the surface of monocytes and if this cellular form can function in extracellular cross-linking reactions. We show for the first time that active FXIII-A is exposed on the surface of IL-4- and IL-10-activated monocytes and that this cellular pool of transglutaminase stabilizes thrombi against fibrinolytic degradation.

## 2. Results

### 2.1. FXIII-A Antigen and Activity Are Exposed on the Surface of Human Monocytes and THP-1 Cells 

FXIII-A is synthesized by monocytes [9,18] but lacks a classical secretion signal and has been assumed to exert only intracellular crosslinking activity. We examined whether FXIII-A is exposed on the surface of human monocytes or THP-1 cells in response to specific stimuli using flow cytometry and confocal microscopy. Human-derived monocytes or THP-1 cells were left unstimulated or were stimulated with IL-4, IL-10 or LPS, as classical monocyte-activating stimuli [19]. 

**Figure 1:**
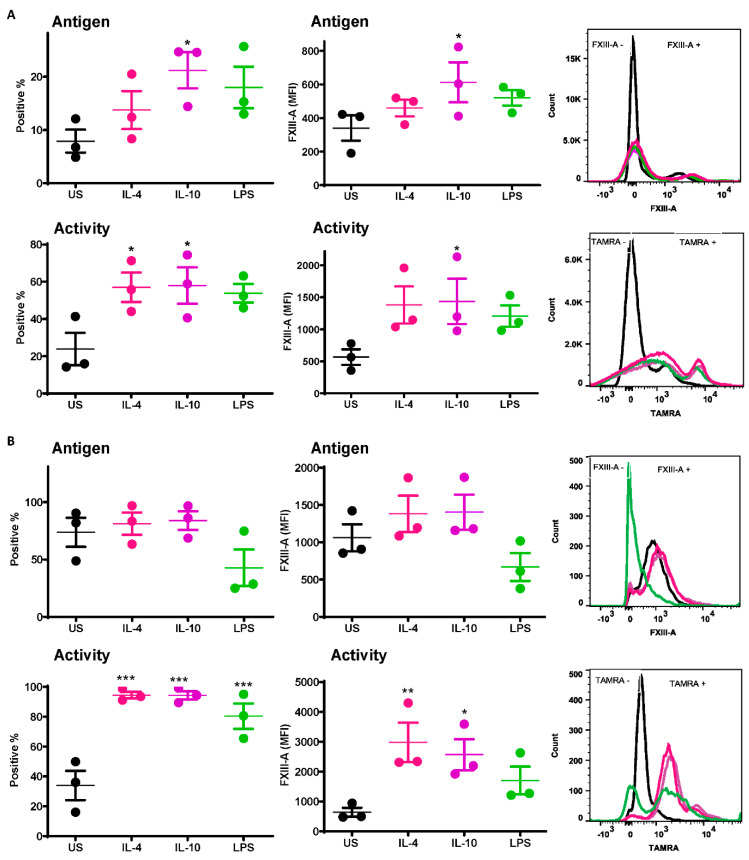
Active FXIII-A is exposed on the surface of isolated human monocytes and THP-1 cells. (**A**) Isolated human monocytes or (**B**) THP-1 cells were left unstimulated or stimulated with IL-4 (20 ng/mL), IL-10 (20 ng/mL) or LPS (100 ng/mL) for 24 h prior to staining using FITC labelled anti-FXIII-A antibody or TAMRA donor FXIII-A substrate for detection of functional activity. Samples were then analyzed using an LSR II flow cytometer. Data are expressed as a percentage of FXIII-A positive monocytes and median fluorescence intensity (MFI) values as the mean ± SEM, *n* = 3. * *p* < 0.05; ** *p* < 0.01; *** *p* < 0.001 vs. resting cells.

A significant increase was observed in the number of IL-10-stimulated monocytes exposing the FXIII-A antigen relative to unstimulated cells (21.2 ± 3.4% vs. 7.9 ± 2.1% *p* < 0.05; Figure 1A). Increased numbers of monocytes exposing the FXIII-A antigen were also observed post-stimulation with IL-4 and LPS (Figure 1A) but did not reach statistical significance. Median fluorescence intensity (MFI) was also significantly increased following stimulation with IL-10 compared to resting cells (613 ± 119 % vs. 340 ± 75% *p* < 0.05; Figure 1A). FXIII-A activity, detected using the TAMRA substrate, was significantly increased with IL-4 and IL-10 compared to resting cells (*p* < 0.05; Figure 1A); however, the MFI was significantly increased with IL-10 only (*p* < 0.05; Figure 1A). These data indicate that basal levels of the FXIII-A antigen on monocytes were increased following stimuli, particularly IL-10, but that functional FXIII-A was significantly elevated post-stimulation. 

The basal level of the FXIII-A antigen on the surface of resting THP-1 cells was almost 10-fold higher than that on human-derived monocytes (73.6 ± 12.7% vs. 7.9 ± 2.1%). Stimulation of THP-1 cells with IL-4 and IL-10 had a negligible impact on the number of cells positive for the FXIII-A antigen or the MFI (Figure 1B). In contrast there was a dramatic increase in THP-1 cells positive for FXIII-A activity post-stimulation with IL-4, IL-10 and LPS compared to unstimulated cells (*p* < 0.001; Figure 1B). However, the MFI was only significantly increased following stimulation with IL-4 and IL-10 (*p* < 0.01; *p* < 0.05; respectively; Figure 1B). These data indicate that despite expression of high basal levels of FXIII-A on THP-1 cells, this pool is largely inactive. Stimulation with IL-4 and Il-10 significantly increased the pool of active FXIII-A on the surface of THP-1. 

**Figure 2:**
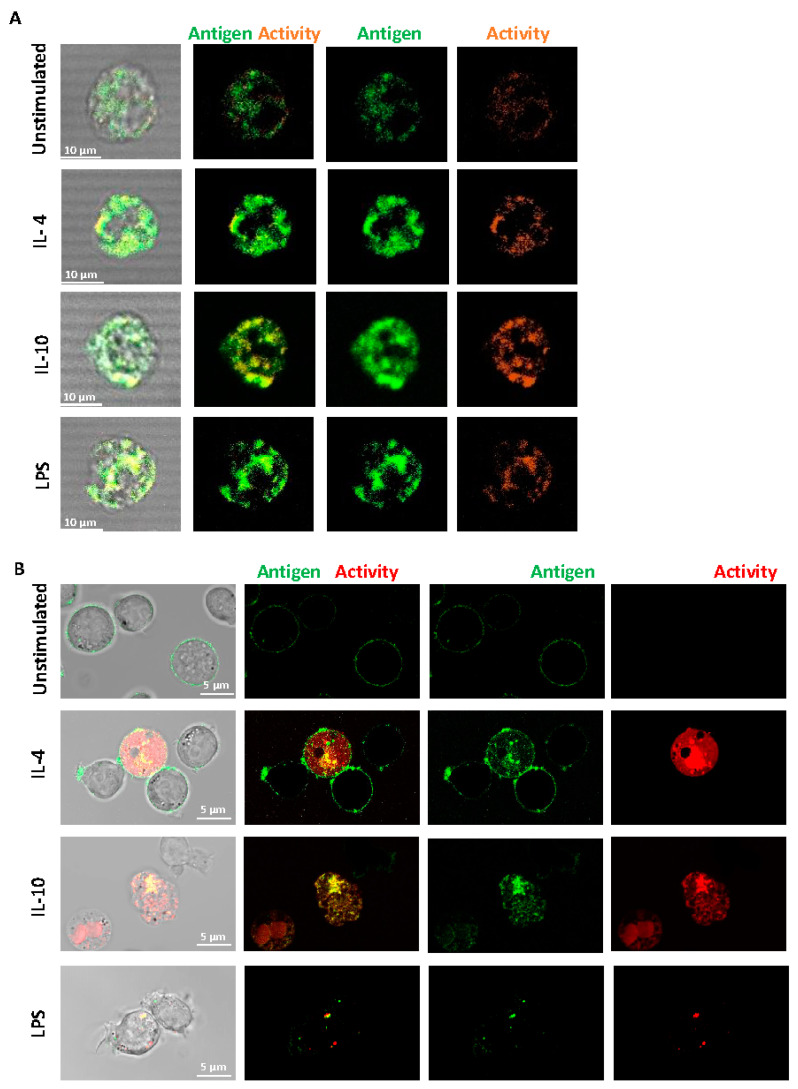
Distribution of FXIII-A on the surface of isolated human monocytes or THP-1 cells. (**A**) Isolated human monocytes or (**B**) THP-1 cells were left unstimulated or stimulated with IL-4 (20 ng/mL), IL-10 (20 ng/mL) or LPS (100 ng/mL) for 24 h. Live cells were stained using FITC labelled anti-FXIII-A antibody or TAMRA donor FXIII-A substrate for detection of activity. Cells were imaged using a LSM880 confocal microscope using a 63 × 1.40 oil immersion. Images are representative of *n* ≥ 3; scale bar 10 μm (**A**) or 5 μm (**B**).

Fluorescence confocal microscopy was performed on live, non-permeabilized cells using a specific antibody to FXIII-A. We found that FXIII-A antigen and activity were expressed on the external membrane of human-derived monocytes (Figure 2A) and were increased post-stimulation. The increased signal was particularly evident with IL-10 stimulation, in line with the flow cytometry data. The FXIII-A antigen and activity were evident on THP-1 cells with an increase evident post-stimulation with IL-4 and IL-10 (Figure 2B). The pattern of staining with IL-10 appeared more diffuse across the THP-1 cells with changes in FXIII-A activity, in agreement with the flow cytometry data (Figure 1B). We investigated a possible synergistic effect of co-stimulation with IL-4 and IL-10 on FXIII-A exposure in THP-1 cells but found no evidence of increased signals over single stimulation (data not shown). Interestingly, stimulation of THP-1 cells with LPS resulted in a different distribution of FXIII-A, evident as a distinct punctate pattern around the periphery of the cells. FXIII-A activity was observed in vesicular compartments within the THP-1 cells. These images suggest that FXIII-A is perhaps differently expressed on THP-1 cells in response to different stimuli. Interestingly, increasing the stimulation time of THP-1 cells to 48 h resulted in a loss of the FXIII-A signal in cells stimulated with LPS, again suggestive of internalization and degradation of FXIII-A. In contrast, a stronger signal was noted with IL-4 and IL-10 following 48 h stimulation (Figure S1). 

### 2.2. FXIII-A Activity in IL-4- or IL-10-Activated THP-1 Cells Primarily Localized to the Cell Membrane

**Figure 3:**
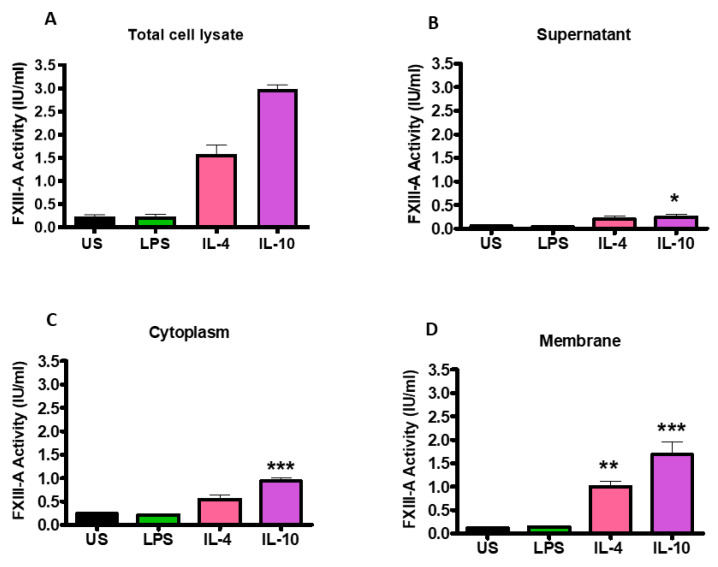
Activation of THP-1 cells by IL-4 or IL-10 increases FXIII-A activity which is primarily associated with the cell cytoplasm and membrane fractions. THP-1 cells (6 × 10⁶ cells/mL) left unstimulated or stimulated with 20 ng/mL IL-4, 20 ng/mL IL-10 or 100 ng/mL LPS for 24 h. The supernatant was collected by centrifugation of cells at 2500× *g* for 5 min. The cells were washed twice in cold sterile PBS and centrifuged at 2500× *g* for 5 min before being resuspended in RIPA buffer. The lysate was centrifuged at 14,000× *g* for 15 min and the supernatant was collected as cytoplasm fractions and the precipitate was resuspended in RIPA buffer as cell membrane fractions. FXIII-A activity (IU/mL) was measured in (**A**) total cell lysate of unstimulated or IL-4, IL-10 or LPS-stimulated cells and cell fractions (**B**) supernatant, (**C**) cytoplasm or (**D**) membrane. * *p* < 0.05; ** *p* < 0.01; *** *p* < 0.001 vs. unstimulated THP-1 cells. Data are expressed as the mean with standard error of the mean SEM (*n =* 3).

Changes in FXIII-A activity following stimulation of THP-1 cells with IL-4, IL-10 and LPS were quantified by an in-house activity assay. Stimulation with IL-4 and IL-10 (1.58 ± 0.20; 2.98 ± 0.10 IU/mL; *p* < 0.001; respectively) significantly increased FXIII-A activity in the total cell lysate compared to unstimulated cells (0.24 ± 0.03; Figure 3A). However, stimulation with LPS did not augment FXIII-A activity, in line with the flow cytometry data. Fractionation of the cells revealed that following stimulation of THP-1 with IL-4 and IL-10, the largest pool of active FXIII-A was located in the membrane (1.00 ± 0.12 IU/mL; *p* < 0.01; 1.68 ± 0.27 IU/mL; *p* < 0.001; respectively; Figure 3D). Active FXIII-A was also detected in the cytoplasm fraction of the cells post-stimulation with IL-4 and IL-10 with a smaller pool within the supernatant. LPS stimulation did not drive up-regulation of FXIII-A in the supernatant, cytoplasm, or membrane fraction. These data indicate that stimulation of THP-1 cells with IL-4 and IL-10 enriches the intracellular pools of the transglutaminase which is directed toward the membrane. 

### 2.3. Monocytes Are Incorporated in Thrombi

**Figure 4:**
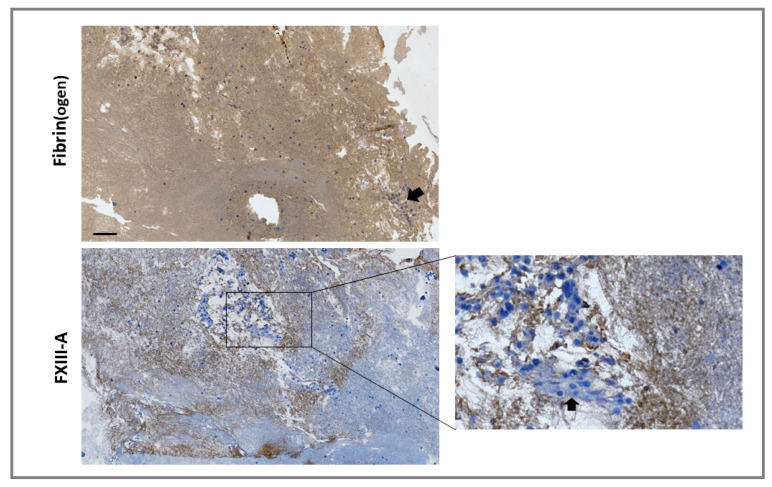
Monocytes are incorporated into forming thrombi. Model thrombi were formed under arterial shear rates with FXIII^–/–^ plasma in the presence of THP-1 cells. Thrombi were fixed in 10% formalin and wax embedded prior to sectioning. Sections were deparaffinized and stained with a polyclonal rabbit anti-fibrin(ogen) (top panel) or anti-FXIII-A (bottom panel) antibodies (brown) and counterstained with Mayer’s haematoxylin (blue) for detection of the nuclei of THP-1 cells. Imaged using 5× magnification. Cell clusters were detected in certain locations (inset and as indicated by arrows). Scale bar = 100 µm.

For externalized monocyte-derived FXIII-A to have a functional impact on clot stability, these cells would need to be incorporated during thrombus formation or have the capacity to invade mature thrombi. We investigated incorporation of the monocytic cell line THP-1 into thrombi under shear stress, using the Chandler model thrombus system, which has previously been used to reveal the role of FXIII-mediated cross-linking in resistance to fibrinolysis [20,21]. Thrombi were formed with FXIII^-/-^-deficient plasma in the presence of THP-1 cells. Immunohistochemical analysis revealed staining for FXIII-A in areas of the thrombi immediately adjacent to cells and in fibrin-rich areas (Figure 4). These results indicate that monocytes act as a vehicle to deliver FXIII-A into thrombi, therefore allowing this transglutaminase to stabilize the forming thrombus against premature fibrinolytic degradation. 

### 2.4. Monocytes Enhance Thrombus Stability in a Transglutaminase-Dependent Manner

**Figure 5:**
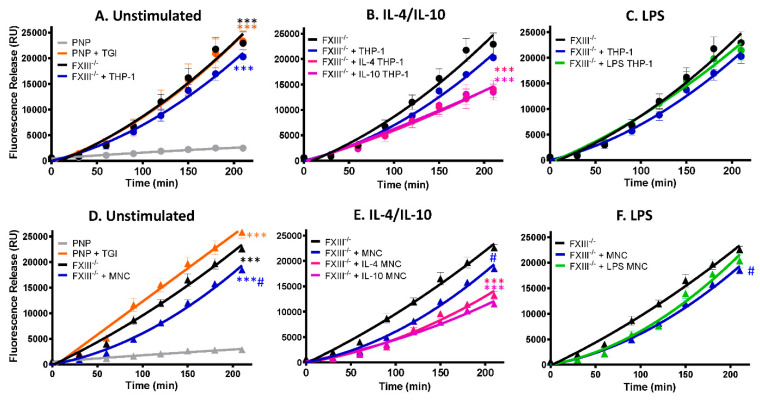
FXIII-A deficient thrombi are stabilized by THP-1 cells and human-derived monocytes stimulated with IL-4 and IL-10. Model thrombi were formed with pooled normal plasma (PNP) or FXIII^-/-^ plasma in the presence of and FITC-labelled fibrinogen under arterial shear rates. Lysis was subsequently induced by tPA (1 μg/mL) and monitored for 4 h. Fluorescence release is directly proportional to the degree of fibrinolysis. PNP thrombi were formed in the absence (grey) and presence (orange) of the TG inhibitor and FXIII^-/-^ thrombi formed without (black) and with (blue) 3 × 10^5^/mL (**A**) THP-1 cells or (**D**) human-derived monocytes. *** *p* < 0.001 vs. PNP; # *p* < 0.05 vs. FXIII^-/-^-deficient thrombi. (**B**) THP-1 cells or (**E**) human-derived monocytes were stimulated for 24 h with IL-4 (pink) or IL-10 (purple) prior to thrombus formation # *p* < 0.05 or *** *p* < 0.001 vs. FXIII^-/-^-deficient thrombi (**C**) THP-1 cells or (**F**) human-derived monocytes were stimulated for 24 h with LPS (green) before addition to thrombi. # *p* < 0.05 vs. FXIII^-/-^-deficient thrombi. Values represent the mean ± SEM, *n* = 4.

We have previously shown that FXIII-A exposed on the surface of activated platelets stabilizes thrombi against fibrinolytic resistance [12]. Here we show that human-derived monocytes and the monocytic cell line, THP-1, expose functional FXIII-A and are incorporated into thrombi under shear conditions. We next investigated whether they could augment thrombus stability. Depletion of plasma FXIII or inhibition of plasma FXIII-A with a TG inhibitor enhanced tPA-mediated lysis >10-fold (Figure 5A, *p* <0.001), consistent with our previous observations [20]. Inclusion of THP-1 cells in model thrombi formed from FXIII deficient plasma did not significantly change the stability against tPA-mediated degradation (Figure 5A). In contrast, THP-1 cells stimulated with IL-4 or IL-10 enhanced thrombus stability 1.7-fold (Figure 5B; *p* < 0.001). These results are consistent with the observed increase in FXIII-A antigen and activity on THP-1 cells. LPS-stimulated THP-1 cells were unable to stabilize thrombi (Figure 5C), reflecting the reduction of the FXIII-A antigen and activity on these cells. 

We next analyzed human-derived monocytes and found unstimulated cells induced a small but significant effect on the stability of thrombi formed from FXIII-deficient thrombi (Figure 5D; *p* < 0.005). IL-4 and IL-10 stimulation of human-derived monocytes enhanced thrombus stability against tPA-mediated degradation approximately 2-fold compared to FXIII deficient thrombi (Figure 5E; *p* < 0.001). In marked contrast, LPS stimulation of monocytes did not significantly impact thrombus stability (Figure 5F). These functional data are in line with the confocal images demonstrating less FXIII-A antigen and activity on unstimulated and LPS-stimulated monocytes. 

**Figure 6:**
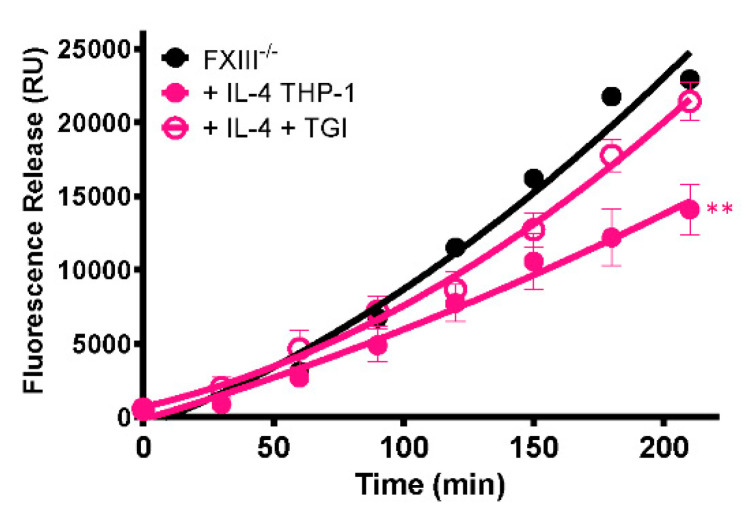
Stabilization of thrombi with IL-4-stimulated THP-1 cells occurs in a transglutaminase-dependent manner. Model thrombi were formed with PNP or FXIII^-/-^ plasma and FITC-labelled fibrinogen under arterial shear rates, and lysis was subsequently induced by tPA (1 μg/mL). Fluorescence release is directly proportional to the degree of fibrinolysis. FXIII^-/-^ thrombi were formed without (black) and with (pink) 3 × 10^5^/mL IL-4 stimulated THP-1 cells in the absence (closed symbols) and presence (open symbols) of the TG inhibitor. Values represent the mean ± SEM, *n* = 4, ** *p* < 0.005 vs. IL-4 stimulated THP-1 cells.

To establish whether the stabilizing effect of cells on thrombi is mediated by FXIII-A, a TG inhibitor was included during thrombus formation. The presence of the TG inhibitor abrogated the stabilizing effect of IL-4-stimulated THP-1 cells (*p* < 0.005; Figure 6), confirming that thrombus resistance is conferred by increased FXIII-A-mediated cross-linking.

## 3. Discussion

Inflammatory cells are now recognized to play a key role in thrombus initiation and propagation [15]. FXIII-A is abundant in monocytes [17] but its potential extracellular role in thrombus formation and stabilization has not been examined. Here we show for the first time that FXIII-A is externalized on the monocyte surface in response to different stimuli. Externalization of this transglutaminase is associated with an increase in thrombus stability, indicating that it is functional in performing extracellular cross-linking reactions. Indeed, staining of FXIII-A was noted within the fibrin mesh of thrombi formed in the presence of monocytes. These data suggest that FXIII-A is constitutively expressed by monocytes and externalized onto the outer leaflet of the membrane, despite the lack of a classical ER release signal and its exclusion from the ER-Golgi pathway in nucleated cells [7]. 

Resting human-derived monocytes demonstrated surface exposure of FXIII-A that was largely inactive in nature. Stimulation of human-derived monocytes with IL-10 and IL-4 increased exposure of FXIII-A which was accompanied by a significant increase in FXIII-A activity and extracellular crosslinking activity. In contrast, stimulation with LPS increased exposure of FXIII-A, as detected by flow cytometry, but had little functional impact on thrombus stability over unstimulated monocytes. In THP-1 cells, surface expression of FXIII-A was around 10-fold higher than human-derived monocytes but was largely inactive. IL-4 and IL-10 stimulation significantly increased FXIII-A activity on the surface of THP-1 cells and in line with this, enhanced thrombus stability. LPS did not alter THP-1 surface expression of FXIII-A to the same degree as human-derived monocytes. The differences in the response of primary monocytes and the monocytic cell line to LPS are documented in the literature and arise due to low levels of CD14 expressed on the immortalized cell line compared to primary cells [22]. Activity assays on THP-1 cells echoed the flow cytometry and confocal data and revealed that FXIII-A is largely associated with the membrane fraction of cells post-stimulation with IL-4 and IL-10. Dual stimulation with IL-4 and IL-10 did not result in a synergistic increase in externalization of FXIII-A on THP-1 cells, suggesting that these activators function through the same pathway to increase externalization of FXIII-A. The number of signaling pathways shared between IL-4 and IL-10 receptors are limited [23], and this knowledge could potentially pinpoint the signaling pathway responsible for externalization of FXIII-A. Exposure of FXIII-A on monocytic cells was time-dependent, with an increase in FXIII-A exposure with IL-4 or IL-10 stimulation over time. In contrast, prolonged stimulation with LPS resulted in a loss of FXIII-A signal, potentially related to internalization. These data are consistent with a previous report showing a time-dependent increase in FXIII-A exposure in monocytes stimulated with IL-4 [24]. Indeed, Törőcsik et. al. suggested that the expression of FXIII-A is an intracellular marker of IL-4-activated macrophages [24] and monocyte-derived dendritic cells also express FXIII-A in response to IL-4 [25]. Monocytes are highly sensitive to IL-10, with the antiflammatory cytokine playing a key role in regulating innate and adaptive immune responses [26]. However, to the best of our knowledge this is the first report that IL-10 can up-regulate monocyte expression of FXIII-A which may function in these processes. 

Expression of FXIII-A was analyzed on non-permeabilized monocytic cells, but FXIII-A and TAMRA staining was observed in a punctuated pattern on LPS-stimulated cells. No such staining was observed in the controls, suggesting this was not accounted for by autofluorescence. Consistent with this, macrophages have been shown to accumulate FXIII-A in pseudopods and cytoplasmic vacuoles [27]. LPS is known to induce vacuolar structures in macrophages that are derived from the plasma membrane and endoplasmic reticulum [28]. In LPS-activated macrophage macropinocytosis occurs constitutively and upon particle phagocytosis, leading to intracellular vacuole formation [29]. Our observations suggest that a similar mechanism of pinocytosis occurs in monocytes in response to LPS stimulation. Interestingly, FXIII-A itself has been implicated in Fcγ-receptor-mediated phagocytosis, with monocytes from FXIII-deficient patients demonstrating decreased phagocytosis compared to healthy controls [30]. 

Cellular FXIII-A has primarily been considered to function in cytoskeletal remodeling as molecules such as myosin [31], actin [32] and vinculin [33] serve as substrates for intracellular FXIII-A. We have previously described a role for platelet-derived FXIII-A in stabilizing thrombi [12]. Here we analyzed the potential function of monocyte-derived FXIII-A in thrombus stability and found that cellular FXIII-A of monocyte origin could act to promote thrombus stability against fibrinolytic degradation. Monocytes were detected in thrombi formed from FXIII-deficient plasma under a physiological flow rate, thus indicating incorporation of cells into forming thrombi and consistent with the other reported roles of monocytes in thrombus initiation and propagation [15]. Addition of IL-4 and IL-10 activated monocytes significantly increased the stability of thrombi formed from FXIII-deficient plasma, whereas inclusion of a transglutaminase inhibitor abrogated the stabilizing effect of IL-4-stimulated monocytes in FXIII-deficient thrombi. In contrast, LPS-stimulated monocytes have no additional impact on thrombus stability over resting cells, most likely due to reduced exposure of functional FXIII-A. The 1,3-dimethy-2-imidazolium derivative used here inactivates FXIII-A [34] but we cannot rule out a contribution of tranglutaminase 2 which is also present in monocytes [35]. However, taken together with the increase in FXIII-A externalization on monocytes in response to IL-4 and IL-10, these data suggest that cellular FXIII-A contributes to extracellular cross-linking reactions.

Monocytes activated by different agonists give rise to different phenotypes and functions [36]. IL-4 results in a reparative phenotype, IL-10 activation leads to development of immunosuppressive function whereas LPS drives proinflammatory responses. IL-10 is increased in the vein wall upon induction of thrombosis and helps to dampen inflammation [37]. Furthermore, recombinant IL-10 inhibits thrombosis in vivo [37] and contrary to this, individuals with depressed levels of IL-10 show enhanced levels of arterial and venous thrombosis [38,39]. These effects could be explained by the ability of IL-10 to directly attenuate monocyte tissue factor exposure and modulate fibrinolysis [40]. Monocyte-derived macrophages within experimental thrombi are primarily of the M2 phenotype [41]. The increase in FXIII-A externalization on monocytes of a reparative phenotype is perhaps not surprising given the now well-documented role of this transglutaminase in wound healing [42]. FXIII-deficient patients and FXIII-A-deficient mice display delayed wound healing, which can be corrected with infusion of FXIII-A [43,44]. High levels of FXIII activity have been observed in healing infarct tissue and deficient mice demonstrate increased cardiac rupture, which is rescued by infusion of FXIII-A_2_B_2_ [45]. Promotion of wound healing of surgical wounds in patients by direct application of FXIII has also been described [1,46]. Interestingly, thrombin treatment of monocytes does not augment exposure of FXIII-A [18], suggesting these cells may contribute to hemostasis in a situation where there is also an increase in the type 2 immune response, for example, in a wound-healing capacity. 

This study is the first to address the direct impact of monocyte-derived FXIII-A on thrombus stability. We have shown that IL-4 or IL-10 stimulation of monocytes promotes externalization and accumulation of active FXIII-A on the cell membrane that enhances thrombus stability for fibrinolytic degradation. Monocytes of reparative/immunosuppressive phenotype may therefore function to stabilize thrombi at sites of inflammation thereby promoting efficient wound healing. These observations on external cross-linking functions of monocyte-derived FXIII-A are particularly relevant in light of the mounting evidence that monocytes are players in venous thrombus formation [15] and can be recruited during venous thrombus formation [16]. In addition to FXIII-A, monocytes harbor procoagulant tissue factor [15] and profibrinolytic proteins, specifically uPA [47]. Given this reservoir of hemostatic proteins which could function in thrombus initiation, stabilization, resolution and wound healing, further work is imperative to delineate the complex role of these inflammatory cells in venous thrombosis. 

## 4. Materials and Methods

### 4.1. Blood Collection and Preparation of Serum

Ethical approval for blood sampling from consenting healthy volunteers was granted by the Ethics Review Board of the College of Life Science & Medicine, University of Aberdeen, in accordance with the declaration of Helsinki. Blood samples were taken by venepuncture and collected into EDTA vacuettes (Greiner Bio-one Ltd., Stonehouse, UK) for monocyte isolate or into serum separating blood tubes. Serum was prepared by incubating at 37 °C for 30 min and then at 4 °C for 30 min. Tubes were centrifuged at 400× *g* for 20 min. Serum was collected and de-complemented by incubating at 56 °C for 30 min before storing at −18 °C. For preparation of platelet-poor plasma, blood was drawn into 3.2% sodium citrate and spun at 1860× *g* for 30 min at 4 °C. Plasma from at least 20 donors was pooled, aliquoted and stored at −70 °C for pooled normal plasma (PNP).

### 4.2. Isolation of Peripheral Blood Mononuclear Cells

Peripheral Blood Mononuclear Cells (PBMCs) were isolated by density gradient centrifugation using Lymphoprep (Axis-Shield, Dundee, UK) according to manufacturers’ recommendation. Monocytes were positively selected from PBMCs by magnetic-activated cell sorting (MACS), using anti-CD14^+^ MicroBeads (Miltenyi Biotec, Surrey, UK). 

### 4.3. Culture and Stimulation of Isolated Monocytes and THP-1 

Isolated human monocytes were allowed to rest for 24 h prior to stimulation in RPMI 1640 Medium (Gibco, ThermoFisher, Paisley, UK) supplemented with 1% L-glutamine (Gibco), 1% penicillin/streptomycin (Gibco) and 5% de-complemented human serum. 

THP-1 cell line (Sigma–Aldrich, St-Louis, MO, USA), a human monocytic cell line derived from monocytic leukemia patients, was cultured in RPMI 1640 supplemented with L-glutamine, 10% fetal calf serum (FCS; VWR International Ltd, Lutterworth, UK) and 1% penicillin and streptomycin (PenStrep) at 37 °C with 5% CO_2_. 

THP-1 or monocytes were seeded into 24-well plates (6 × 10^5^cells/mL) in RPMI 1640 and were left untreated or were treated with 20 ng/mL interleukin-4 (IL-4; Fischer Scientific, Loughborough, UK), 20 ng/mL interleukin-10 (IL-10; PeproTech, London, UK) or 100 ng/mL lipopolysaccharide (LPS; Sigma–Aldrich) for 24 h or in some experiments for the time of 48 h. Cells were washed following stimulation to ensure serum was removed.

### 4.4. Preparation of THP-1 Cell Fractions 

THP-1 cells (6 × 10^5^cells/mL) were subjected to centrifugation of cells at 2500× *g* for 5 min to collect the supernatant. The cells were washed twice in cold sterile PBS (Sigma–Aldrich) and centrifuged at 2500× *g* for 5 min before resuspending in RIPA buffer (ThermoFisher) and incubating for 15 min on ice. The lysate was centrifuged at 14,000× *g* for 15 min and the supernatant was collected as cytoplasm fractions. The precipitate was resuspended to the original volume in RIPA buffer reflecting the membrane fraction.

### 4.5. Flow Cytometry 

Stimulated monocytes/THP-1 cells were re-suspended in Hanks balanced salt solution (HBSS) containing 1% BSA (bovine serum albumin) for 1 h at ambient temperature and incubated with a FITC labelled anti-FXIII-A antibody (40 μg/mL; Zedira GmBH, Darmstadt, Germany) or the fluorescent amine donor substrate, TAMRA (N-tetramethylrhodaminylcadaverine; 40 μg/mL; Zedira) with 0.5 mM CaCl_2_ for 30 min. Cells were then washed by centrifuging at 300× *g* in HBSS twice to remove unbound antibody or substrate. FXIII-A exposure and activity were detected using an LSR II flow cytometer (Becton Dickinson, Franklin Lakes, NJ, USA). A minimum of 10,000 events were collected. Data analysis was performed using FlowJo software (Tree Star Inc, BD, Ashland, OR, USA.) and gated based on a forward and side scatter gate. 

### 4.6. Fluorescent Confocal Microscopy 

Cells were harvested, centrifuged and re-suspended in Hanks balanced salt solution (HBSS) containing 1% BSA (bovine serum albumin) for 1 h at ambient temperature. An FITC labelled polyclonal anti-FXIII-A antibody or TAMRA (40 μg/mL) supplemented with 0.5 mM CaCl_2_ was added for 30 min before washing twice with HBSS. Cells were added to Ibidi μ-slide VI^0.4^ chambers and imaged using brightfield and at excitation wavelengths of 488 nm or 547 nm on a Zeiss LSM 880 confocal microscope with a 63 × 1.40 oil immersion objective or with a 60 × objective on an EVOS fluorescence microscope and analyzed by using Zen 2012 software.

### 4.7. FXIII Activity Assay 

FXIII activity in unstimulated and stimulated THP-1 cells was quantified using an in-house activity assay as previously described [12,21]. Briefly, human fibronectin (5 µg/well) was used to coat a 96-well plate (CoStar, Corning, MA, USA). Recombinant FXIII-A (Tretten; Novo Nordisk A/S, Denmark) and samples were preactivated with 1 U/mL bovine thrombin (Sigma Aldrich) for 5 min at 37 °C and residual thrombin was neutralized by the addition of 20 U/mL hirudin (Sigma Aldrich). A standard curve was prepared by diluting standard FXIIIa in 0.1 M Tris, 1 mM Dithiothreitol (pH 7.4). The transglutaminase reaction, in 0.1 M Tris, 1 mM Dithiothreitol, 1 % BSA, 0.5 mM 5-(biotinamido) pentylamine (ThermoFisher) and 5 mM CaCl_2_, was stopped after 2 h at 37 °C by the addition of 2 mM EDTA in 0.1 M Tris (pH 7.4). Plates were then washed and blocked with 0.5 % (*w*/*v*) milk powder for 30 min at 37 °C and incorporated biotinylated amine was detected using *para*-nitrophenyl phosphate substrate. The absorbance was read at 405 nm (reference filter 690 nm) on a Labsystems iEMS 1401 spectrophotometer (Labsystems Oy, Helsinki, Finland). 

### 4.8. Thrombus Formation, Lysis and Fixation

Model thrombi were formed as described [12]. Briefly, PNP or factor XIII-deficient plasma (FXIII^–/–^; Affinity Biologicals, Ancaster, Canada) containing 45 µg/mL FITC (fluorescein isothiocyanate) labelled fibrinogen was re-calcified with CaCl_2_ (10.9 mM). Stimulated monocytes were included at 3 × 10^5^ cells/mL^.^ In some experiments, 1 mM non-reversible inhibitor of TG (1,3-dimethyl-2-[(2-oxopropyl) thio] imidazolium chloride; produced in house) was included. Thrombi were extracted from the loops and bathed in 1 µg/mL tissue plasminogen activator (tPA; Genetech, CA, USA). Samples were taken at 30-min intervals for 4 h. Fluorescence was read (excitation 485 nm and emission 528 nm) on a Biotek Instruments Fluorometer (Agilent Technologies LDA UK Ltd, Harwell, UK).

Alternatively, thrombi, formed in the absence of FITC-fibrinogen, were fixed in 10% formalin for 24 h before embedding in wax and sectioning.

### 4.9. Immunohistochemistry

Sections were deparaffinized and dehydrated followed by microwaving in citrate buffer (pH 6.0) for antigen retrieval and endogenous peroxidase activity quenched with 3% (vol/vol) H2O2 in methanol. Sections were stained with polyclonal rabbit, anti-human antibodies for fibrinogen (Agilent Technologies) and FXIII-A (Abcam, Cambridge, UK) and subsequently with biotin-conjugated mouse secondary antibody (Vector laboratories, CA, USA). Avidin/Biotin ABC enzyme complex was applied, and slides were incubated with ImmPACT DAB substrate (Vector laboratories). Slides were counterstained with Mayer’s hematoxylin and nuclei blued with Scots tap water. Sections were imaged using a Zeiss Z1 Axioscan slide scanner at ×5 and ×20 magnification.

### 4.10. Data Analysis

Rates of lysis (FU/min^−1^) for model thrombi were determined by best fit of the slope to a centered second-order polynomial quadratic in GraphPad Prism 5.04 and used to calculate fold differences in lysis. Statistical analysis was performed on lysis data using one-way repeated measurement ANOVA (analysis of variance) followed by Dunnett’s multiple comparison post hoc test in GraphPad Prism 5.04 (CA, USA). P-values less than 0.05 were taken as statistically significant, and values are given as the mean ± SEM (standard error of mean).

## Data Availability

The data presented in this study are available on request from the corresponding author.

## Supplementary Materials

The following are available online at https://www.mdpi.com/article/10.3390/ijms22126591/s1.

## References

[1] Muszbek L., Yee V.C., Hevessy Z. (1999). Blood coagulation factor XIII: Structure and function. Thromb. Res..

[2] Muszbek L., Bereczky Z., Bagoly Z., Komaromi I., Katona E. (2011). Factor XIII: A coagulation factor with multiple plasmatic and cellular functions. Physiol. Rev..

[3] Dardik R., Solomon A., Loscalzo J., Eskaraev R., Bialik A., Goldberg I., Schiby G., Inbal A. (2003). Novel proangiogenic effect of factor XIII associated with suppression of thrombospondin 1 expression. Arter. Thromb. Vasc. Biol..

[4] Alshehri F.S.M., Whyte C.S., Mutch N.J. (2021). Factor XIII-A: An Indispensable “Factor” in Haemostasis and Wound Healing. Int. J. Mol. Sci..

[5] Polgar J., Hidasi V., Muszbek L. (1990). Non-proteolytic activation of cellular protransglutaminase (placenta macrophage factor XIII). Biochem. J..

[6] Muszbek L., Polgar J., Boda Z. (1993). Platelet factor XIII becomes active without the release of activation peptide during platelet activation. Thromb. Haemost..

[7] Kaetsu H., Hashiguchi T., Foster D., Ichinose A. (1996). Expression and release of the a and b subunits for human coagulation factor XIII in baby hamster kidney (BHK) cells. J. Biochem..

[8] Cordell P.A., Kile B.T., Standeven K.F., Josefsson E.C., Pease R.J., Grant P.J. (2010). Association of coagulation factor XIII-A with Golgi proteins within monocyte-macrophages: Implications for subcellular trafficking and secretion. Blood.

[9] Poon M.C., Russell J.A., Low S., Sinclair G.D., Jones A.R., Blahey W., Ruether B.A., Hoar D.I. (1989). Hemopoietic origin of factor XIII A subunits in platelets, monocytes, and plasma. Evidence from bone marrow transplantation studies. J. Clin. Investig..

[10] Inbal A., Muszbek L., Lubetsky A., Katona E., Levi I., Karpati L., Nagler A. (2004). Platelets but not monocytes contribute to the plasma levels of factor XIII subunit A in patients undergoing autologous peripheral blood stem cell transplantation. Blood Coagul. Fibrinolysis.

[11] Beckers C.M.L., Simpson K.R., Griffin K.J., Brown J.M., Cheah L.T., Smith K.A., Vacher J., Cordell P.A., Kearney M.T., Grant P.J. (2017). Cre/lox Studies Identify Resident Macrophages as the Major Source of Circulating Coagulation Factor XIII-A. Arterioscler. Thromb. Vasc. Biol..

[12] Mitchell J.L., Lionikiene A.S., Fraser S.R., Whyte C.S., Booth N.A., Mutch N.J. (2014). Functional factor XIII-A is exposed on the stimulated platelet surface. Blood.

[13] Stalker T.J., Welsh J.D., Tomaiuolo M., Wu J., Colace T.V., Diamond S.L., Brass L.F. (2014). A systems approach to hemostasis: 3. Thrombus consolidation regulates intrathrombus solute transport and local thrombin activity. Blood.

[14] Smeeth L., Cook C., Thomas S., Hall A.J., Hubbard R., Vallance P. (2006). Risk of deep vein thrombosis and pulmonary embolism after acute infection in a community setting. Lancet.

[15] von Bruhl M.L., Stark K., Steinhart A., Chandraratne S., Konrad I., Lorenz M., Khandoga A., Tirniceriu A., Coletti R., Kollnberger M. (2012). Monocytes, neutrophils, and platelets cooperate to initiate and propagate venous thrombosis in mice in vivo. J. Exp. Med..

[16] Laurance S., Bertin F.R., Ebrahimian T., Kassim Y., Rys R.N., Lehoux S., Lemarie C.A., Blostein M.D. (2017). Gas6 Promotes Inflammatory (CCR2(hi)CX3CR1(lo)) Monocyte Recruitment in Venous Thrombosis. Arterioscler. Thromb. Vasc. Biol..

[17] Muszbek L., Adany R., Szegedi G., Polgar J., Kavai M. (1985). Factor XIII of blood coagulation in human monocytes. Thromb. Res..

[18] Kradin R.L., Lynch G.W., Kurnick J.T., Erikson M., Colvin R.B., Mcdonagh J. (1987). Factor-Xiii-a Is Synthesized and Expressed on the Surface of U937-Cells and Alveolar Macrophages. Blood.

[19] Ivanova E.A., Orekhov A.N. (2016). Monocyte Activation in Immunopathology: Cellular Test for Development of Diagnostics and Therapy. J. Immunol. Res..

[20] Fraser S.R., Booth N.A., Mutch N.J. (2011). The antifibrinolytic function of factor XIII is exclusively expressed through alpha-antiplasmin cross-linking. Blood.

[21] Mutch N.J., Koikkalainen J.S., Fraser S.R., Duthie K.M., Griffin M., Mitchell J., Watson H.G., Booth N.A. (2010). Model thrombi formed under flow reveal the role of factor XIII-mediated cross-linking in resistance to fibrinolysis. J. Thromb. Haemost. JTH.

[22] Bosshart H., Heinzelmann M. (2016). THP-1 cells as a model for human monocytes. Ann. Transl. Med..

[23] Tugal D., Liao X., Jain M.K. (2013). Transcriptional control of macrophage polarization. Arterioscler. Thromb. Vasc. Biol..

[24] Torocsik D., Bardos H., Nagy L., Adany R. (2005). Identification of factor XIII-A as a marker of alternative macrophage activation. Cell Mol. Life Sci..

[25] Grassi F., Dezutter-Dambuyant C., McIlroy D., Jacquet C., Yoneda K., Imamura S., Boumsell L., Schmitt D., Autran B., Debre P. (1998). Monocyte-derived dendritic cells have a phenotype comparable to that of dermal dendritic cells and display ultrastructural granules distinct from Birbeck granules. J. Leukoc. Biol..

[26] Sabat R., Grutz G., Warszawska K., Kirsch S., Witte E., Wolk K., Geginat J. (2010). Biology of interleukin-10. Cytokine Growth Factor Rev..

[27] Adany R., Antal M. (1996). Three different cell types can synthesize factor XIII subunit A in the human liver. Thromb. Haemost..

[28] Yoshida K., Ono M., Sawada H. (1999). Lipopolysaccharide-induced vacuoles in macrophages: Their origin is plasma membrane-derived organelles and endoplasmic reticulum, but not lysosomes. J. Endotoxin. Res..

[29] Wong A.O., Marthi M., Mendel Z.I., Gregorka B., Swanson M.S., Swanson J.A. (2018). Renitence vacuoles facilitate protection against phagolysosomal damage in activated macrophages. Mol. Biol. Cell.

[30] Sarvary A., Szucs S., Balogh I., Becsky A., Bardos H., Kavai M., Seligsohn U., Egbring R., Lopaciuk S., Muszbek L. (2004). Possible role of factor XIII subunit A in Fcgamma and complement receptor-mediated phagocytosis. Cell Immunol..

[31] Cohen I., Young-Bandala L., Blankenberg T.A., Siefring G.E., Bruner-Lorand J. (1979). Fibrinoligase-catalyzed cross-linking of myosin from platelet and skeletal muscle. Arch Biochem. Biophys..

[32] Cohen I., Blankenberg T.A., Borden D., Kahn D.R., Veis A. (1980). Factor XIIIa-catalyzed cross-linking of platelet and muscle actin. Regulation by nucleotides. Biochim. Biophys. Acta.

[33] Asijee G.M., Muszbek L., Kappelmayer J., Polgar J., Horvath A., Sturk A. (1988). Platelet vinculin: A substrate of activated factor XIII. Biochim. Biophys. Acta.

[34] Freund K.F., Doshi K.P., Gaul S.L., Claremon D.A., Remy D.C., Baldwin J.J., Pitzenberger S.M., Stern A.M. (1994). Transglutaminase inhibition by 2-[(2-oxopropyl)thio]imidazolium derivatives: Mechanism of factor XIIIa inactivation. Biochemistry.

[35] Sun H., Kaartinen M.T. (2018). Transglutaminase activity regulates differentiation, migration and fusion of osteoclasts via affecting actin dynamics. J. Cell Physiol..

[36] Murray P.J. (2017). Macrophage Polarization. Annu. Rev. Physiol..

[37] Downing L.J., Strieter R.M., Kadell A.M., Wilke C.A., Austin J.C., Hare B.D., Burdick M.D., Greenfield L.J., Wakefield T.W. (1998). IL-10 regulates thrombus-induced vein wall inflammation and thrombosis. J. Immunol..

[38] Caligiuri G., Rudling M., Ollivier V., Jacob M.P., Michel J.B., Hansson G.K., Nicoletti A. (2003). Interleukin-10 deficiency increases atherosclerosis, thrombosis, and low-density lipoproteins in apolipoprotein E knockout mice. Mol. Med..

[39] Poredos P., Jezovnik M.K. (2011). In patients with idiopathic venous thrombosis, interleukin-10 is decreased and related to endothelial dysfunction. Heart Vessel..

[40] Pajkrt D., van der Poll T., Levi M., Cutler D.L., Affrime M.B., van den Ende A., ten Cate J.W., van Deventer S.J. (1997). Interleukin-10 inhibits activation of coagulation and fibrinolysis during human endotoxemia. Blood.

[41] Gallagher K.A., Obi A.T., Elfline M.A., Hogikyan E., Luke C.E., Henke S., Coleman D., Henke P.K. (2016). Alterations in macrophage phenotypes in experimental venous thrombosis. J. Vasc. Surg. Venous Lymphat. Disord..

[42] Soendergaard C., Kvist P.H., Seidelin J.B., Nielsen O.H. (2013). Tissue-regenerating functions of coagulation factor XIII. J. Thromb. Haemost. JTH.

[43] Vanscheidt W., Hasler K., Wokalek H., Niedner R., Schopf E. (1991). Factor XIII-deficiency in the blood of venous leg ulcer patients. Acta Derm Venereol..

[44] Inbal A., Lubetsky A., Krapp T., Castel D., Shaish A., Dickneitte G., Modis L., Muszbek L., Inbal A. (2005). Impaired wound healing in factor XIII deficient mice. Thromb. Haemost..

[45] Nahrendorf M., Hu K., Frantz S., Jaffer F.A., Tung C.H., Hiller K.H., Voll S., Nordbeck P., Sosnovik D., Gattenlohner S. (2006). Factor XIII deficiency causes cardiac rupture, impairs wound healing, and aggravates cardiac remodeling in mice with myocardial infarction. Circulation.

[46] Gierhake F.W., Papastavrou N., Zimmermann K., Bohn H., Schwick H.G. (1974). Prophylaxis of post-operative disturbances of wound healing with factor XIII substitution (author’s transl). Dtsch Med Wochenschr..

[47] Humphries J., Gossage J.A., Modarai B., Burnand K.G., Sisson T.H., Murdoch C., Smith A. (2009). Monocyte urokinase-type plasminogen activator up-regulation reduces thrombus size in a model of venous thrombosis. J. Vasc. Surg..

