# Supplementary material for: Monocytes Expose Factor XIII-A and Stabilize Thrombi against Fibrinolytic Degradation
Source: Int J Mol Sci. 2021 Jun 19;22(12):6591. doi: 10.3390/ijms22126591 (PMC8234680; doi:10.3390/ijms22126591)
Supplement: Supplementary file 1 [file ijms-22-06591-s001.zip › ijms-1250036-supplementary.pdf]

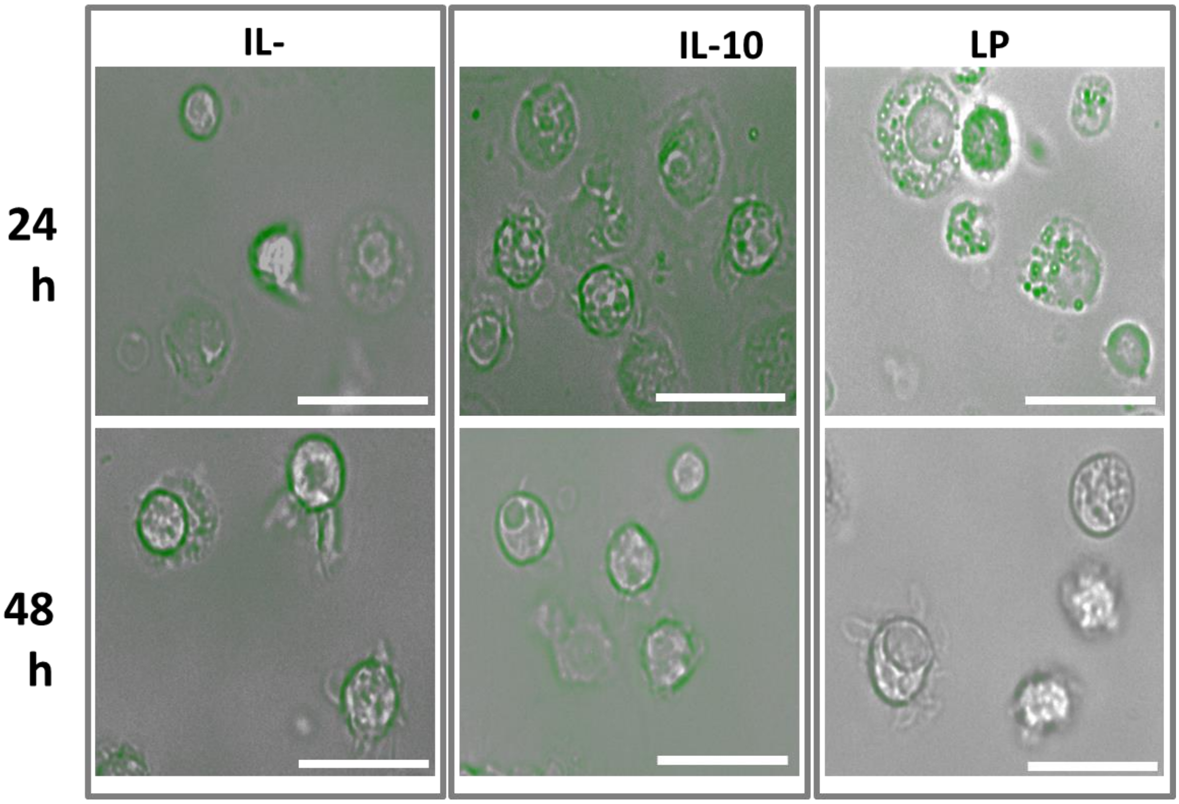

Figure S1. Differences in FXIII-A externalization in THP-1 cells over time. THP-1 cells were stimulated with IL-4 (20 ng/ml), IL-10 (20 ng/ml) or LPS (100 ng/ml) for 24 h or 48 h. Live cells were stained using FITC labelled anti FXIIIa antibody and imaged using an EVOS fluorescence microscope at x 60 oil objective (Scale bar = 25 µm). Images are representative of $n \geq 3$.
